# A Retrospective Assessment of Laboratory Findings and Cytokine Markers in Severe SARS-CoV-2 Infection among Patients of Roma Population

**DOI:** 10.3390/jcm11226777

**Published:** 2022-11-16

**Authors:** Alexandra Mocanu, Voichita Elena Lazureanu, Adelina Raluca Marinescu, Talida Georgiana Cut, Ruxandra Laza, Laura-Cristina Rusu, Adina Maria Marza, Andreea Nelson-Twakor, Rodica Anamaria Negrean, Irina-Maria Popescu, Alexandru Ovidiu Mederle

**Affiliations:** 1 Department XIII, Discipline of Infectious Diseases, “Victor Babes” University of Medicine and Pharmacy Timisoara, Eftimie Murgu Square 2, 300041 Timisoara, Romania; 2 Department of Oral Pathology, Multidisciplinary Center for Research, Evaluation, Diagnosis and Therapies in Oral Medicine, “Victor Babes” University of Medicine and Pharmacy Timisoara, Eftimie Murgu Square 2, 300041 Timisoara, Romania; 3 Department of Surgery, Multidisciplinary Center for Research, Evaluation, Diagnosis and Therapies in Oral Medicine, “Victor Babes” University of Medicine and Pharmacy Timisoara, Eftimie Murgu Square 2, 300041 Timisoara, Romania; 4 Faculty of Medicine and Pharmacy, “Ovidius” University of Constanta, 900527 Constanta, Romania; 5 Faculty of Medicine and Pharmacy, University of Oradea, 410073 Oradea, Romania; 6 Department XIII, Discipline of Epidemiology, “Victor Babes” University of Medicine and Pharmacy Timisoara, Eftimie Murgu Square 2, 300041 Timisoara, Romania

**Keywords:** COVID-19, SARS-CoV-2, inflammatory markers, cytokine storm, disease severity

## Abstract

Growing research data suggests that the severity of COVID-19 is linked with higher levels of inflammatory mediators, such as cytokines, chemokines, tumor necrosis factor, C-reactive protein, ferritin, and D-dimers. In addition, it was evident from the existing research data that the severity of SARS-CoV-2 infection differs according to independent risk factors such as race and ethnicity. Some scarce evidence shows that the European Roma community is likely to be at an elevated risk of illness and death during the pandemic due to their lifestyle, social factors, and economics. Assuming that precautions must be taken to protect this population from coronavirus infections and from widening existing disparities in comparison with the Romanian ethnic population, the current study aimed to observe the clinical evolution of the Roma patients with severe SARS-CoV-2 infection in correlation with the laboratory findings and inflammatory markers involved. After calculating the sample size requirements, we included 83 Roma patients admitted to the hospital with severe COVID-19 and 236 patients of Romanian ethnicity with the same inclusion criteria. Patients were selected from the period stretching from March 2020 to December 2021, before COVID-19 vaccines were introduced. Compared with the general population, the Roma patients with severe SARS-CoV-2 infection had a higher unemployment rate (39.8%), and most of them were residing in rural regions (65.4%). There were significantly more overweight patients in the Roma group than in the control group (57.8% vs. 40.7%), and it was also observed that high blood pressure and diabetes mellitus were significantly more prevalent in the Roma patients. They had significantly longer mean duration of hospitalization was significantly longer in the group of Roma patients (18.1 days vs. 16.3 days). IL-6 and CRP levels were significantly more elevated during admission in the group of Roma patients (43.4% vs. 28.4%); however, IL-6 levels normalized at discharge, but ESR remained high. Although ICU admissions were significantly more frequent in this group, the mortality rate was not significantly higher than in the general population. It is necessary to plan different healthcare strategies aimed at special populations, such as the Roma ethnicity to prevent disparities in negative outcomes reflected in this study. The results imply that community-health collaborations between organizations of minority groups and healthcare professionals can mitigate the disproportionate consequences of the pandemic on Roma.

## 1. Introduction

After being discovered for the first time in December 2019 in Wuhan City, which is located in Hubei Province in China, the SARS-CoV-2 virus quickly spread all over the globe, igniting the still ongoing pandemic [1,2,3]. The most common symptoms of COVID-19 are mostly non-specific, and they include high prevalence rates of fever, tiredness, and dry cough, while pneumonia, thrombo-embolic events, and acute respiratory distress syndrome (ARDS) are all outcomes and possible severe manifestations of severe COVID-19 that is likely to be triggered by the so-called “cytokine storm” [4,5].

The cytokine storm syndrome is one of the most dreaded and serious complications that can occur in COVID-19 patients, being caused by an overactive immune response to the virus, which is triggered by inflammatory cell infiltration in the lungs, activation of T-helper 1 reactions, and abundant release of proinflammatory cytokines into the circulation [6]. The cytokine storm syndrome was responsible for multiple organ dysfunction syndromes and disseminated intravascular coagulation, while the majority of investigations have shown the presence of venous thromboembolism and microthrombi in arterioles and venules in COVID-19 patient corpses [7,8]. Therefore, the prompt treatment of this cytokine storm in its early stage, with the use of immunomodulators, corticosteroids, and cytokine antagonists, is suggested by some experts to be the most important factor in lowering the death rate of these individuals and determining fewer intensive care unit admissions [9,10]. Every patient group is at risk of contracting SARS-CoV-2, and similarly, the cytokine storm and ARDS may develop in any patient with COVID-19, despite the fact that a number of known and unknown variables might impact the severity of the result [11,12,13].

According to the findings of a number of studies, the Roma community is likely to be at an increased risk of contracting illnesses and dying as well as suffering psychological, social, and economic repercussions as a result of the COVID-19 pandemic [14]. In addition, some racial and ethnic groups seem to be more prone to comorbidities that predispose them to worse COVID-19 outcomes, as well as certain specific genetic variables that connect the severity of infection with their demographic characteristic. The Roma population is one of the most underprivileged and undeveloped minority groups on the European continent [15]. Roma communities were previously described to exhibit different health outcomes, including a far shorter life expectancy, a higher frequency of both physical and mental health issues, and a greater adoption of dangerous health behaviors. From a syndemic perspective, it is believed that Roma populations exhibit worse health outcomes due to an association of genetic differences, lifestyle factors, and social underdevelopment. According to one study, there are also much higher rates of substance use among Roma populations, while contagious illnesses, such as measles, hepatitis, and tuberculosis, disproportionately affect them due to their lifestyle pattern of living in close and numerous family units, as well as their beliefs regarding conventional medicine [16].

Similarly, it is hypothesized that among a community that has not been immunized against SARS-CoV-2, Roma patients would have a more severe reaction to the viral infection [17,18]. However, to the best of our knowledge, there are not a lot of data available on the dynamics of SARS-CoV-2 viral manifestations in the Roma community. As a result, the purpose of this research was to investigate the clinical progression of Roma patients who were severely infected with SARS-CoV-2 in association with the laboratory results and inflammatory markers that were involved.

## 2. Materials and Methods

### 2.1. Study Design and Ethics

Patients were enrolled in the current observational retrospective study if their hospital admission occurred between March 2020 and December 2020, representing the beginning of the COVID-19 pandemic in Romania, respectively, the beginning of the COVID-19 vaccination campaign. The research was carried out at the “Victor Babes” University of Medicine and Pharmacy from Timisoara in the Department of Infectious Disease of the “Victor Babes” Hospital for Infectious Disease and Pneumology. As a retrospective research, data was collected from the paper records and digital records of patients diagnosed during the study period with severe SARS-CoV-2 infection.

The infectious disease clinic affiliated with the “Victor Babes” University of Medicine and Pharmacy, as an auxiliary of the “Victor Babes” Hospital for Infectious Disease and Pneumology from Timisoara, operates under the laws of the local commission of ethics that approves scientific research that functions in accordance with the International Conference on Harmonization from Helsinki regarding technical requirements for registration of pharmaceuticals for human use. The research complied with the ethics criteria from the university where the study was developed and was approved by the ethics committee of both institutions on 28 May 2021, with approval number 5058.

### 2.2. Inclusion Criteria and Variables

A database and patient paper record search were conducted to determine the number of adult Roma patients admitted to the hospital with severe SARS-CoV-2 infection. Patients were included if they matched the following criteria: (1) being older than 18 years; (2) if their paper records mentioned the Roma ethnicity; (3) being unvaccinated for COVID-19 to avoid biased results from vaccines [19]. The vaccination status was checked based on the QR code certificate issued in Romania, as part of the European Union regulations during the COVID-19 pandemic; and (4) being admitted with a severe form of COVID-19. The severe SARS-CoV-2 infection was considered as patients who meet any of the following criteria [20,21,22,23]: (a) presenting to the hospital with respiratory distress syndrome or respiratory rates higher than 30/min; (b) the finger oxygen saturation measured after 5 min of rest was lower than 93%; (c) PaO_2_ (the arterial oxygen partial pressure)/FiO_2_ (the inspired oxygen fraction) ≤ 300 mmHg; and (d) affected lung area on computed tomography (CT) of more than 50%. The COVID-19 status was defined by a positive polymerase chain reaction test (PCR) from oropharyngeal and nasal swabs [24]. A predefined patient personal form was used to gather demographic, clinical, and outcome data from electronic medical records, as well as to identify the ethnicity of the patients.

Using a convenience sample method, we estimated that at least 78 patients from the Roma population should be included in the analysis to provide proper statistical power. The sample size was calculated for a Roma population proportion in Romania of about 3%, according to the most recent census [25]. Other considerations for the sample size were a 99% confidence level and a 5% margin of error. A total of 83 patients from the Roma population were included in the analysis to match the inclusion criteria. A secondary cohort of 236 patients with severe COVID-19 was included in the analysis from the Romanian ethnicity to provide an accurate comparison group.

The variables taken into consideration included background data (age, gender, area of residence, occupation, body mass index, smoking status, alcohol use), the presence of chronic comorbidities (high blood pressure, lung disease, diabetes mellitus, cerebrovascular disease, digestive and liver problems, kidney disease, depression, malignancy), and COVID-19 transmission source. COVID-19 data that was analyzed comprised signs and symptoms, COVID-19 patient outcomes, and COVID-19 treatment. Based on the existing national guidelines [26,27], clinical picture, and documented comorbidities, COVID-19 patients received antiviral agents (Favipiravir, Remdesivir), broad-spectrum antibiotics in case of suspicioning or confirming an infection, anticoagulant treatment, steroids, and immune modulators for the duration of hospital admission. Lastly, the laboratory data were included, comprising patients’ complete blood count, liver function indicators, renal function parameters, lipid profile, and the following inflammatory markers: procalcitonin, D-dimers, IL-6, TNF-alpha, ferritin, ESR, CRP, and fibrinogen.

### 2.3. Statistical Analysis

IBM SPSS v.27 and MedCalc v.20 were used for statistical analysis. We calculated the absolute (n) and relative (%) frequencies of categorical variables and compared their proportions using Chi-square and Fisher’s exact test. After testing the available data for normality with the Shapiro-Wilk test, we used the Mann-Whitney test to compare non-Gaussian variables, and we reported them by the median and interquartile range (IQR). The mean and standard deviation of continuous variables with a normal distribution were compared using the student’s *t*-test (unpaired, independent samples). A significance level of 0.05 was chosen as the alpha value.

## 3. Results

### Patients’ Background Characteristics

A total of 319 patients with severe SARS-CoV-2 infection were included in the study, where 83 of them were of Roma ethnicity, and the other 236 were Romanians. The baseline characteristics between patients of Roma and Romanian ethnicity with severe COVID-19 are presented in Table 1. It was observed that the age groups were significantly different since admitted Roma patients with severe COVID-19 were younger than the patients in the control group (38.6% older than 65 years vs. 48.3% in the other group, *p*-value = 0.019). There were significantly more overweight patients in the Roma group than in the control group (57.8% vs. 40.7%, *p*-value = 0.023). Other significant changes were the area of residence and occupation, where there were more unemployed Roma patients, and most of them lived in the rural region (55.4%). It was also observed that high blood pressure and diabetes mellitus were significantly more prevalent in the group of Roma patients (44.6% vs. 32.2%, *p*-value = 0.042), respectively 38.6% vs. 22.9% (*p*-value = 0.005).

Table 2 presents a comparison of SARS-CoV-2 infection signs, symptoms, and outcomes between patients of Roma and Romanian ethnicity with severe COVID-19. There were no obvious differences in the prevalence of signs and symptoms between study groups, as well as there was no difference in the treatment approach during the hospital admission of the cohort of patients included in this study. However, it was observed that the mean duration of hospitalization was significantly longer in the group of Roma patients (18.1 days vs. 16.3 days, *p*-value = 0.016). There were also more ICU admissions and requirements of oxygen supplementation in patients of Roma ethnicity with severe SARS-CoV-2 infection (44.6% vs. 31.8%, *p*-value = 0.035), respectively 75.9% vs. 64.0% (*p*-value = 0.046). Finally, the mortality rate was not significantly influenced when comparing the two groups.

The comparison of laboratory findings at admission between patients of Roma and Romanian ethnicity with severe COVID-19 is presented in Table 3. It was observed that the complete blood count was generally altered but without statistically significant differences between the two groups of patients with severe SARS-CoV-2 infection. Regarding liver function, we observed that fasting glucose levels were significantly more elevated in the group of Roma patients, with 43.4% of patients having higher than normal glucose levels, compared with 28.8% in the control group (*p*-value = 0.014). Creatinine levels at admission were also higher in the Roma population (39.8% values outside the normal range), as well as cholesterol levels were more altered (38.6% vs. 25.4%, *p*-value = 0.023). Similarly, the median values of laboratory findings between the comparison groups showed significant differences between the fasting glucose levels, creatinine, and cholesterol levels (Table 3).

**Figure 1:**
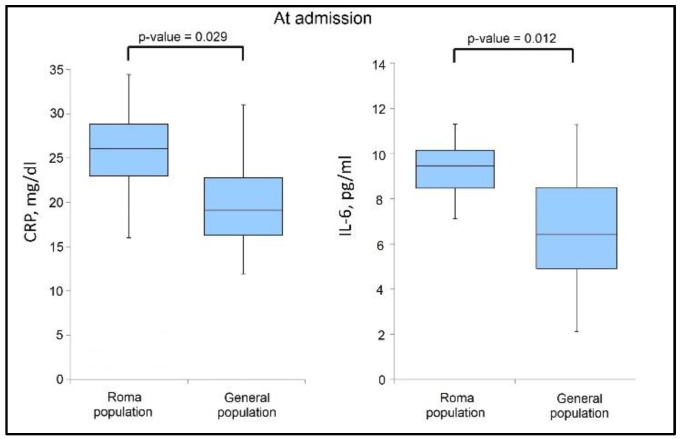
Boxplot of significant findings in biological parameters at admission.

**Figure 2:**
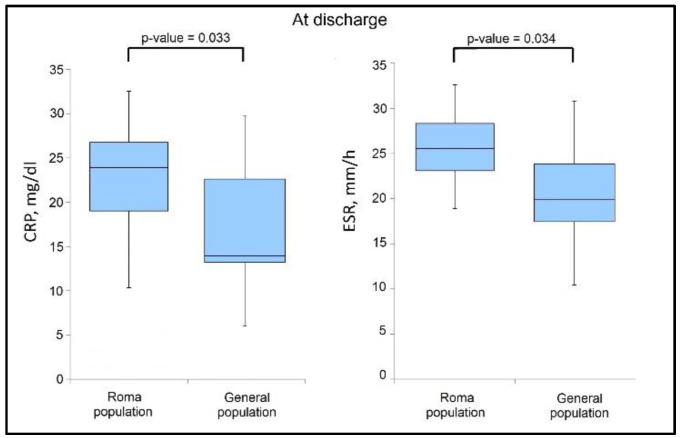
Boxplot of significant findings in biological parameters at discharge.

The analysis of cytokine levels and inflammatory markers presented in Table 4 shows that IL-6 and CRP levels were significantly more elevated during admission in the group of Roma patients (43.4% vs. 28.4%, *p*-value = 0.012), respectively 63.9% vs. 50.0% (*p*-value = 0.029), as presented in Figure 1. However, it was observed that at discharge (Figure 2) that the IL-6 levels normalized when comparing the two study groups, although CRP levels remained significantly higher in the group of Roma patients (47.6% vs. 32.8%, *p*-value = 0.033). ESR levels were also more elevated in the Roma patient group versus the general population, with 54.0% of patients having out-of-range ESR values, compared to 38.9% (*p*-value = 0.034), as seen in Table 5.

## 4. Discussion

### 4.1. Literature Findings

One of the most marginalized groups in Europe is the Roma community, which numbers between 10 and 15 million people and is comprised of a variety of ethnic groups with nomadic ways of life. Despite this, the Roma people account for more than 3% of the permanently established population in Romania. Besides the risks experienced by the Roma people as a community at a public health level, there are still no studies analyzing the pathophysiology of SARS-CoV-2 infection in this particular ethnic group [28].

Compared to the overall population of patients with severe SARS-CoV-2 infection, there were substantially more obese patients in the Roma group than in the control group (57.8% vs. 40.7%), and high blood pressure and diabetes mellitus were also much more frequent in the Roma patients. In the group of Roma patients, the average length of stay was much longer (18.1 days vs. 16.3 days). IL-6 and CRP levels were considerably higher in Roma patients at admission (43.4% vs. 28.2%); however, IL-6 levels decreased at discharge while ESR remained increased. Although ICU hospitalizations were much greater in this group, the death rate was comparable to that of the overall population of hospitalized patients with severe COVID-19. Since the patients included in our study represented severe SARS-CoV-2 infections, there was frequent use of antivirals and broad-spectrum antibiotic therapy due to a higher rate of complications, similar to what was described in other studies [29].

Although no studies were reported before the specifical findings regarding the inflammatory markers in patients of the Roma population with severe COVID-19, it was previously discussed how IL-6 levels could indicate a persistent lung injury in patients with SARS-CoV-2 infection [30,31]. Notably, these studies described how individuals with persistent pulmonary lesions, as determined by high CT scores, had elevated IL-6 levels at discharge and follow-up period, which can also explain why our group of patients with higher IL-6 at admission had more ICU admission and requirements for oxygen supplementation. Importantly, the peak expression of IL-6 prior to the deterioration of lung damage was mostly seen in patients with persistent lesions, and multivariate analysis revealed that IL-6 level at admission was an independent predictor associated with persistent pulmonary injury [32]. Thus, this significantly higher proportion of Roma patients with severe COVID-19 that showed high levels of IL-6 at admission can explain why these patients also had continuously elevated inflammatory markers at admission, such as CRP and ESR.

Another possible explanation for increased levels of inflammatory markers in the group of Roma patients presented in the current study is the correlation with the higher proportion of diabetes mellitus, obesity, and dyslipidemia in these patients. Several investigations revealed that the levels of inflammatory biomarkers rose with the degree of obesity, and a high correlation was found between the biomarkers and BMI [33,34]. Recent research suggests that higher plasma levels of inflammatory markers are related to an increased risk of dyslipidemia. Infiltration of inflammatory cells into adipose tissue may affect adipocyte lipid and cytokine production, which may have downstream implications on lipid metabolism disease. Metabolic syndrome was associated with the elevation of type 1 T helper cells in conjunction with many cytokines, including IL-6 and TNF [35]. IL-6 is a pleiotropic cytokine that promotes the creation of acute phase proteins, such as reactive C protein, amyloid A, and fibrinogen, in the acute inflammatory response during infections and posttraumatic tissue damage. This acute phase reaction is accompanied by an increase in blood viscosity and platelet activation. High plasma fibrinogen levels lead to a decline in serum HDL-cholesterol levels.

High ESR levels at discharge were also reported in other cases of patients with COVID-19. It was described that ESR started to grow dramatically about two weeks after the first positive SARS-CoV-2 test. The elevated level of ESR persisted despite the disappearance of fever and dry cough, the improvement of chest CT findings, and the negative results of a PCR test [36]. The overproduction of inflammatory cytokines in severe cases of SARS-CoV-2 infection may be related to the increased levels of CRP that are seen in these patients. Cytokines are what defend the body against microorganisms, but when the immune system is overactive, they may cause irreversible harm to the lung tissue. Therefore, the generation of CRP is stimulated by inflammatory cytokines as well as by the breakdown of tissue in individuals who have COVID-19. To summarize, an increased level of CRP may be a useful early marker in predicting the probability of disease progression in non-severe individuals who have COVID-19. This may enable medical professionals to identify such people at an early stage so that they can begin early therapy. In addition, COVID-19 patients who have increased levels of CRP need constant monitoring and therapy even if they did not acquire symptoms that fit the criteria for the severe disease course [37]. For instance, the severe instances were shown to have greater levels of C-reactive protein when compared to the moderate cases in each and every one of the studies that were reviewed in a recently published systematic review, wherein 78.7% of the severe COVID-19 cases, there was a substantial rise in C-reactive protein levels [38].

The Roma population is one of the most underrepresented minorities on the continent [28]. Roma communities have radically inferior health outcomes, such as a much lower life expectancy, an increased incidence of both physical and mental health issues, and greater adoption of dangerous health behaviors. Roma communities also have significantly higher rates of substance abuse and unhealthy nutritional habits [39]. Furthermore, Roma groups are disproportionately affected by communicable diseases such as measles, hepatitis, and tuberculosis. Additionally, Roma groups are less likely to engage with healthcare services, including primary and preventive care such as vaccinations, child health, and maternal care, due to barriers including culture, language, and health literacy [40].

In our study, Roma patients had a higher unemployment rate (39.8%), and most of them were residing in rural regions (65.4%). Because of these variables, Roma populations are disproportionately exposed to COVID-19 hazards, including the lack of proper access to healthcare services when facing a severe infection, the risk of developing a condition that requires treatment, and the risk of transmission within their communities. Living circumstances also make it more difficult for vulnerable populations to participate in mitigation efforts, such as regularly washing their hands, maintaining a physical distance, and gaining access to medical treatment [41]. The enhanced dangers posed by COVID-19 for these communities have the potential to make the health inequalities that already exist among Roma groups much worse and to have an effect on the health of the general population. In accordance with these hypotheses, the current study determined significant changes in patients of Roma ethnicity that were admitted to hospitals with severe SARS-CoV-2 infection during the COVID-19 pandemic in Romania.

Considering that many of the Roma people are migrants or seasonal workers around Europe, while those residing in Romania have higher rates of unemployment, they risk facing disproportionate social care and medical care, which can place them at increased risk of COVID-19 morbidity and mortality. Besides those who are unemployed, the Roma people occupy more low-income jobs, while it is considered that workers from low-income occupations and minority groups are employed in occupations that put them at greater risk of exposure to COVID-19 than other workers. Low-income workers may face financial disincentives for absence even if they are sick or vulnerable, including the reluctance to wear masks and maintain proper hygiene conditions, increasing the workplace transmission of the SARS-CoV-2 virus [42].

### 4.2. Study Limitations

Although the selected cohort satisfied the sample size requirements for statistical power, the number of patients is still not big enough to identify COVID-19 outcomes that occur with a lower incidence. Therefore, it is recommended that further studies that include a larger population size of Roma patients are necessary to understand the bigger picture of this community. Also, including only patients with severe SARS-CoV-2 infection didn’t allow for a proper understanding of the proportion of severe infections in the Roma community compared with the country average. It is worth mentioning the risk of selection bias that might occur in this study since all patients were admitted to a tertiary hospital. Therefore, it is likely that the presented cases of COVID-19 can be of greater severity than the average population. Another drawback of retrospective cohort studies is that since several medical practitioners were engaged in patient care, the assessment of risk variables and outcomes throughout the database is likely to be less precise and consistent than in prospective cohort research.

## 5. Conclusions

Being from the Roma ethnic group determines a higher likelihood of severe complications necessitating ICU admission during SARS-CoV-2 infection. IL-6 and CRP levels were significantly more elevated during admission in the Roma group, which might correlate with the higher proportion of patients being sent to the ICU. Remarkably, ICU admissions were significantly more frequent among Roma patients with severe COVID-19, but the mortality rate was not significantly higher than in the control group representing the general population with severe COVID-19. Among survivors, IL-6 levels normalized at discharge, but ESR remained elevated and significantly higher than the control group. Although several inflammatory markers were more elevated than in the general population of patients with severe COVID-19, people of the Roma minority suffer from a higher prevalence of associated comorbidities that can contribute to these negative outcomes and higher ICU admissions. Efforts should prioritize expanding access to health services and health information and be carried out in an equitable, culturally sensitive, and non-discriminatory manner utilizing evidence-based strategies such as dedicated services, health worker outreach, and specialist roles for community leaders. Further studies with larger cohorts are needed to understand if Roma ethnicity is a risk factor for COVID-19 severity and mortality and to describe the evolution and outcomes of mild and moderate-severity SARS-CoV-2 infection in this ethnic group.

## Figures and Tables

**Table 1:** Comparison of baseline characteristics between patients of Roma and Romanian ethnicity with severe COVID-19.

Variables *	Roma (*n* = 83)	Romanian (*n* = 236)	*p*-Value
**Background data**			
Age			0.019
18–40 years	14 (16.9%)	16 (6.8%)	
40–65 years	37 (44.6%)	106 (44.9%)	
>65 years	32 (38.6%)	114 (48.3%)	
**Sex**			0.185
Men	53 (63.9%)	131 (55.5%)	
Women	30 (36.1%)	105 (44.5%)	
**BMI**			0.023
Underweight (<18.5 kg/m^2^)	4 (4.8%)	12 (5.1%)	
Normal weight (18.5–25.0 kg/m^2^)	31 (37.3%)	128 (54.2%)	
Overweight (>25.0 kg/m^2^)	48 (57.8%)	96 (40.7%)	
**Other characteristics**			
Area of residence (urban)	37 (44.6%)	144 (61.0%)	0.009
Occupation (unemployed)	33 (39.8%)	52 (22.0%)	0.001
Relationship status (married)	80 (96.4%)	211 (89.4%)	0.053
**Substance use behavior**			
Chronic smoking	26 (31.3%)	63 (26.7%)	0.418
Chronic alcohol use	5 (6.0%)	12 (5.1%)	0.743
**Chronic comorbidities**			
High blood pressure	37 (44.6%)	76 (32.2%)	0.042
Lung	15 (18.1%)	29 (12.3%)	0.188
Diabetes mellitus	32 (38.6%)	54 (22.9%)	0.005
Cerebrovascular	20 (24.1%)	36 (15.3%)	0.068
Digestive & liver	11 (13.3%)	19 (8.1%)	0.162
Kidney	14 (16.9%)	29 (12.3%)	0.293
Depression	2 (2.4%)	16 (6.8%)	0.137
Malignancy	4 (4.8%)	17 (7.2%)	0.451
Other	6 (7.2%)	20 (8.5%)	0.721
**COVID-19 transmission source**			0.537
Community	9 (10.8%)	26 (11.0%)	
Family	29 (34.9%)	98 (41.5%)	
Unknown source	45 (54.2%)	112 (47.5%)	

* Data reported as *n* (%) and calculated using the Chi-square test and Fisher’s exact unless specified differently; BMI—Body Mass Index.

**Table 2:** Comparison of SARS-CoV-2 infection signs, symptoms, and outcomes between patients of Roma and Romanian ethnicity with severe COVID-19.

Variables *	Roma (*n* = 83)	Romanian (*n* = 236)	*p*-Value
**Signs and Symptoms**			
Cough	52 (62.7%)	160 (67.8%)	0.393
Fever	59 (71.1%)	173 (73.3%)	0.695
Dyspnea	43 (51.8%)	127 (53.8%)	0.752
Headache	10 (12.0%)	38 (16.1%)	0.374
Digestive symptoms	21 (25.3%)	43 (18.2%)	0.165
Anosmia/ageusia	24 (28.9%)	71 (30.1%)	0.841
Fatigue	72 (86.7%)	194 (82.2%)	0.338
Myalgia/arthralgia	22 (26.5%)	61 (25.8%)	0.906
Dysphagia	4 (4.8%)	13 (5.5%)	0.809
**COVID-19 treatment**			
Antivirals	69 (83.1%)	201 (85.2%)	0.657
Corticosteroids	65 (78.3%)	19 (83.5%)	0.291
Antibiotics	70 (84.3%)	209 (88.6%)	0.317
Anticoagulant	61 (73.5%)	175 (74.2%)	0.906
Immune modulators	23 (27.7%)	62 (26.3%)	0.798
**COVID-19 Outcomes**			
Mean duration of hospital stay	18.1 ± 5.3	16.3 ± 6.0	0.016
Median duration from symptom onset until hospital admission	4 [1–6]	5 [1–7]	0.590
Viral clearance	16.5 ± 6.6	14.9 ± 6.8	0.064
ICU admission	37 (44.6%)	75 (31.8%)	0.035
Median duration of ICU stays	8 [2–14]	5 [1–9]	<0.001
Severe in-hospital complications	16 (19.3%)	34 (14.4%)	0.293
Oxygen supplementation	63 (75.9%)	151 (64.0%)	0.046
Mortality	20 (24.1%)	38 (16.1%)	0.104

* Data reported as *n* (%) and calculated using Chi-square test and Fisher’s exact unless specified differently; BMI—Body Mass Index; ICU—Intensive Care Unit.

**Table 3:** Comparison of laboratory findings at admission between patients of Roma and Romanian ethnicity with severe COVID-19.

Variables *	Normal Range	Roma (*n* = 83)	Romanian (*n* = 236)	*p*-Value
**Complete blood count**				
RBC (millions/mm^3^)	4.35–5.65	5.8 (3.1)	5.7 (3.3)	0.798
PLT (thousands/mm^3^)	150–450	336 (129)	319 (107)	0.188
WBC (thousands/mm^3^)	4.5–11.0	12.4 (4.6)	12.6 (4.7)	0.906
Neutrophils (thousands/mm^3^)	1.5–8.0	9.0 (3.8)	8.8 (3.5)	0.289
Lymphocytes (thousands/mm^3^)	1.0–4.8	6.2 (2.2)	6.6 (2.9)	0.296
Hemoglobin (g/dL)	13.0–17.0	14.1 (5.0)	14.5 (5.2)	0.687
Hematocrit (%)	36–48	39 (9)	40 (11)	0.267
**Liver function**				
Fasting glucose (mmol/L) ^	60–125	122 (51)	97 (43)	0.014
ALT (U/L)	7–35	37 (12)	34 (11)	0.354
AST (U/L)	10–40	38 (8)	33 (8)	0.422
LDH (U/L)	140–280	240 (44)	246 (47)	0.791
PT (seconds)	11.0–13.5	12.6 (4.0)	12.9 (4.1)	0.673
**Renal function**				
Creatinine (µmol/L) ^	0.74–1.35	1.49 (0.72)	1.22 (0.66)	0.031
BUN (mmol/L)	2.1–8.5	8.5 (3.1)	7.8 (3.2)	0.140
eGFR	>60	64 (25)	60 (22)	0.128
**Lipid profile**				
Total cholesterol (mg/dL) ^	100–200	247 (103)	214 (96)	0.023
Triglycerides	50–150	152 (39)	148 (40)	0.105
LDL-C (mg/dL)	<100	94 (50)	91 (47)	0.464
HDL-C (mg/dL)	40–60	38 (26)	40 (29)	0.251

* Data reported as median (IQR) unless specified differently; IQR—Interquartile Range; ^ Below the statistical significance threshold (0.05) using the Mann-Whitney *U*-test; WBC—White Blood Cells; RBC—Red Blood Cells; AST—Aspartate Aminotransferase; ALT—Alanine Aminotransferase; eGFR—Estimated Glomerular Filtration Rate; LDH—Lactate Dehydrogenase; BUN—Blood Urea Nitrogen; PT—Prothrombin Time; LDL—Low-Density Lipoproteins; HDL—High-Density Lipoproteins.

**Table 4:** Comparison of cytokines and inflammatory markers at admission between patients of Roma and Romanian ethnicity with severe COVID-19.

Variables *	Normal Range	Roma (*n* = 83)	Romanian (*n* = 236)	*p*-Value
Procalcitonin (ug/L)	0–0.25	0.94 (0.69)	0.81 (0.53)	0.635
D-dimers (ng/mL)	<250	308 (166)	311 (169)	0.853
IL-6 (pg/mL) ^	0.8–6.4	9.2 (3.7)	6.9 (4.8)	0.012
TNF-α (pg/mL)	7.8–12.2	14.0 (6.4)	14.9 (6.8)	0.233
Ferritin (ng/mL)	20–250	272 (92)	247 (62)	0.417
ESR (mm/h)	0–22	34 (24)	28 (13)	0.057
CRP (mg/dL) ^	0–10	25 (16)	19 (9)	0.029
Fibrinogen (g/L)	2–4	6.1 (3.3)	5.8 (3.0)	0.276

* Median (IQR) unless specified differently; IQR—Interquartile Range; ^ Below the statistical significance threshold (0.05) using the Mann-Whitney *U*-test; CRP—C-reactive Protein; IL—Interleukin; TNF—Tumor Necrosis Factor; IFN—Interferon; ESR—Erythrocyte Sedimentation Rate.

**Table 5:** Comparison of cytokines and inflammatory markers at discharge between patients of Roma and Romanian ethnicity with severe COVID-19.

Variables *	Normal Range	Roma (*n* = 83)	Romanian (*n* = 236)	*p*-Value
Procalcitonin (ug/L)	0–0.25	0.66 (0.38)	0.57 (0.33)	0.216
D-dimers (ng/mL)	<250	240 (93)	246 (98)	0.833
IL-6 (pg/mL)	0.8–6.4	7.4 (3.9)	7.6 (3.7)	0.744
TNF-α (pg/mL)	7.8–12.2	12.5 (4.0)	12.6 (4.2)	0.882
Ferritin (ng/mL)	20–250	228 (72)	214 (66)	0.491
ESR (mm/h) ^	0–22	26 (16)	20 (11)	0.034
CRP (mg/dL) ^	0–10	27 (9)	15 (14)	0.033
Fibrinogen (g/L)	2–4	4.1 (2.7)	4.8 (3.0)	0.350

* Median (IQR) unless specified differently; IQR—Interquartile Range; ^ Below the statistical significance threshold (0.05) using the Mann-Whitney *U*-test; CRP—C-reactive Protein; IL—Interleukin; TNF—Tumor Necrosis Factor; IFN—Interferon; ESR—Erythrocyte Sedimentation Rate.

## Data Availability

Data available on request.
